# Overcoming diagnostic pitfalls in primary gastric squamous cell carcinoma: the imperative of adequate sampling in an elderly female patient, case report

**DOI:** 10.3389/fonc.2025.1695030

**Published:** 2025-11-17

**Authors:** Wanhui Dong, Li Cheng, Jing Xu, Sheng Xu, Yuling Yin, Qingming Sun, Yong Wu, Yuling Leng

**Affiliations:** 1 Lu’an Hospital Affiliated to Anhui University of Chinese Medicine, Lu’an, China; 2 Hospital Affiliated to Shandong University of Traditional Chinese Medicine, Jinan, China; 3 The Second Affiliated Hospital of Anhui University of Traditional Chinese Medicine, Hefei, China

**Keywords:** gastric squamous cell carcinoma, diagnosis, immunohistochemistry, misdiagnosis, palliative treatment, immunotherapy

## Abstract

**Objective:**

To report the diagnosis and treatment of a rare case of primary gastric squamous cell carcinoma (PGSCC) in an elderly female, and to explore the diagnostic pitfalls and individualized treatment strategies.

**Methods:**

We conducted a retrospective analysis of the clinical, imaging, endoscopic, and pathological data of a 77-year-old female patient. The evolution of the diagnosis from the initial “possible adenocarcinoma” to the final diagnosis of “ squamous cell carcinoma” was emphasized.

**Results:**

The patient presented with abdominal pain. The first endoscopic biopsy, due to the limited tissue sample, showed immunohistochemistry results of CKpan (+), CK7(+), MUC5AC (focal+), and Ki-67(partly+), favoring a diagnosis of adenocarcinoma. A subsequent deep biopsy with complete immunohistochemistry (CK5/6+, P40+, P63+) confirmed the diagnosis of gastric squamous cell carcinoma. Given the patient’s advanced age, poor physical condition, and refusal of surgery, a palliative treatment plan of “Raltitrexed + Sintilimab” was finally decided upon after a multidisciplinary consultation.

**Conclusion:**

This case highlights the importance of adequate sampling in the diagnosis of gastric squamous cell carcinoma to avoid misdiagnosis due to limited specimens. It also provides a potential treatment option of immune therapy combined with low-dose chemotherapy for elderly patients with gastric squamous cell carcinoma who are not suitable for surgery.

## 1 Introduction

**G**astric cancer, as the fifth most common malignant tumor worldwide, has a diverse range of pathological types that directly affect clinical decision-making and patient prognosis (1, 2). Among them, gastric adenocarcinoma is the absolute dominant type, while gastric squamous cell carcinoma (PGSCC) is an extremely rare subtype, with an incidence rate of less than 0.1% (3). Previously, it was believed that gastric squamous cell carcinoma might originate from squamous metaplasia of the gastric mucosa, abnormal differentiation of pluripotent stem cells, or the extension of esophageal squamous epithelium into the stomach (4). Notably, in contrast to gastric adenocarcinoma, which has a clear male predominance (male-to-female ratio of approximately 2:1) (5), PGSCC lacks a clear gender predisposition due to the scarcity of global data. This makes the diagnosis of this elderly female patient particularly valuable for reporting.

The diagnosis of PGSCC faces multiple challenges (6). In terms of pathological morphology, when biopsy specimens are limited, its cellular features can be easily confused with poorly differentiated adenocarcinoma or adenosquamous carcinoma (7). In immunohistochemical discrimination, the full expression of squamous differentiation markers (such as p40 and p63) depends on sufficient and well-preserved tissue samples. A common clinical dilemma is that the initial biopsy may be insufficient in depth or breadth, leading to tissue fragmentation or the acquisition of only necrotic/inflammatory areas. Studies have shown that the uncertainty of small specimen diagnosis is relatively high, especially in elderly patients, where the complexity of diagnosis further increases due to the frequent presence of mucosal atrophy and intestinal metaplasia (8). The ambiguous conclusion of “tendency towards adenocarcinoma” after the first biopsy in this case was precisely due to this - the limited specimen size restricted the complete interpretation of immunohistochemistry, leaving the diagnosis in a dilemma.

There is a significant gap in current research: data on the diagnosis and treatment of PGSCC in elderly women is extremely scarce, and this group has special considerations in terms of physiological status, comorbidity spectrum, and treatment tolerance. The value of this case lies not only in its rarity but also in its complete presentation of a diagnostic shift from “suspected adenocarcinoma” to “confirmed squamous cell carcinoma”.

## 2 Case presentation

**Figure 1:**
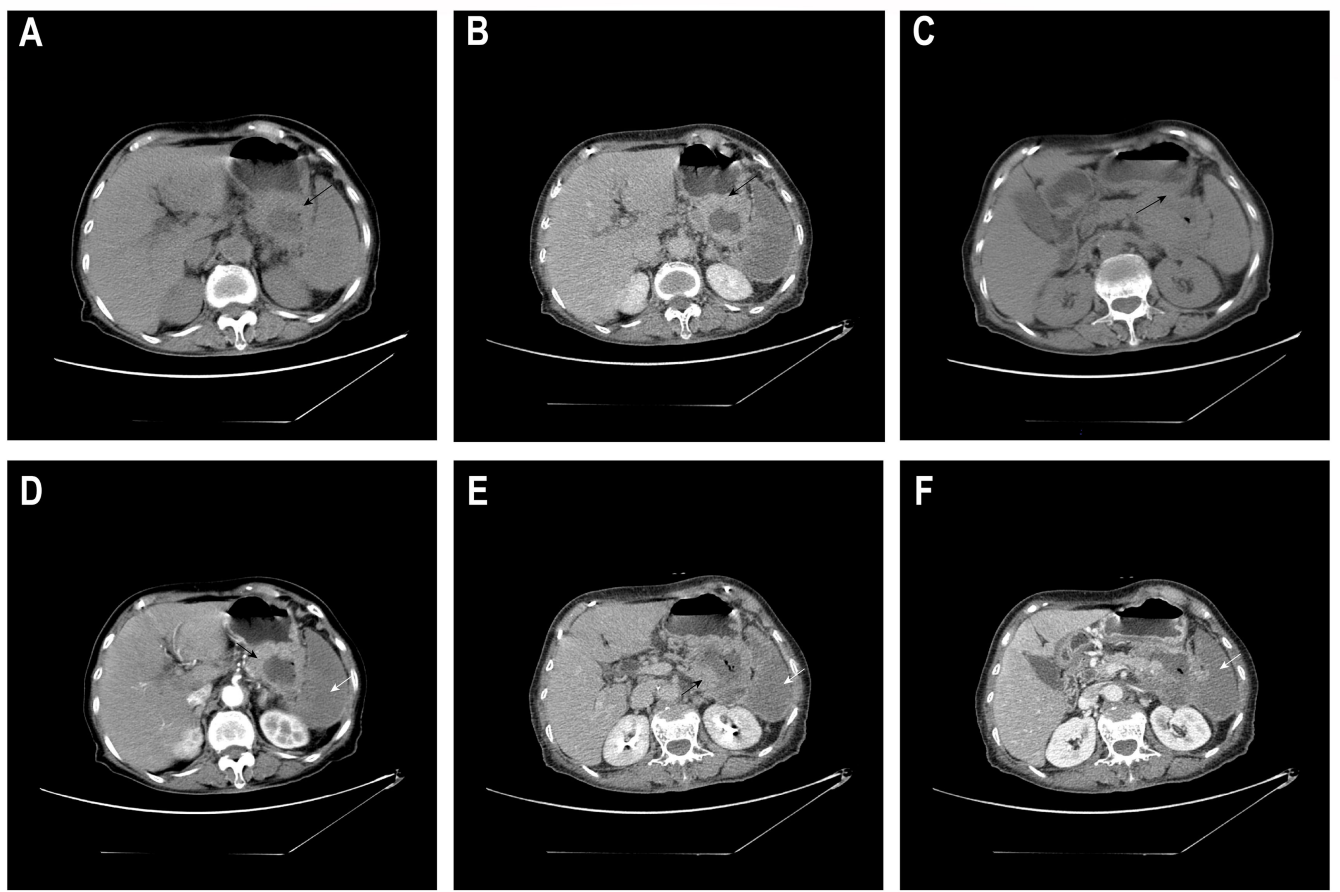
Abdominal CT plain scan plus enhancement. The same axial slice 1 **(A, D, B, E)**: (**A**, plain scan) A space-occupying lesion in the space between the spleen and stomach (black arrow ←); (**D**, arterial phase) The lesion shows significant enhancement at the margin, accompanied by patchy non-enhanced areas in the spleen (white arrow ←); (**B**, venous phase) The lesion shows moderate to high enhancement at the margin (black arrow ←), while the low-density area inside remains non-enhanced; (**E**, equilibrium phase) The spleen is full, with large areas of low/no enhancement (white arrow ←). The same axial slice 2 **(C, F)**: (**C**, plain scan) A low-density space-occupying lesion in the space between the spleen and stomach, with unclear boundaries from the adjacent posterior wall of the stomach, spleen, pancreas and part of the intestinal tract, seemingly communicating with the gastric cavity (black arrow ←); (**F**, venous phase) Large areas of low/no enhancement in the spleen (white arrow ←).

A 77-year-old female patient has experienced mild abdominal pain since early June 2025, mainly in the upper left abdomen. The pain slightly eased after she self-medicated with ibuprofen, but it persisted and was accompanied by irregular fever, with the highest temperature reaching 39 degrees Celsius. On June 2, 2025, at 1 AM, the patient’s abdominal pain intensified. A contrast-enhanced CT scan of the abdomen (**Figure 1**) revealed: 1. A mass-like lesion in the space between the stomach and the spleen, with a local area seemingly communicating with the gastric cavity (suspected to be a malignant lesion of the gastric wall with adjacent tissue invasion)?. Splenic infarction. It is recommended to combine gastroscopy and MRI examination to assist in diagnosis. 2. Small nodules in the liver with enhancement, slight dilation of some intrahepatic bile ducts: small stones in the right kidney: peritoneal effusion and a small amount of pelvic effusion. 3. Chronic bronchitis and emphysema with a few chronic inflammatory lesions: multiple nodular shadows in both lungs. Admitted to the hospital from the emergency department. The patient has a history of hypertension for more than 10 years, taking amlodipine besylate dispersible tablets 5mg once a day; self-reported history of cerebral infarction for more than 10 years, previously taking aspirin 100mg once a day, but stopped due to skin bleeding, and now changed to clopidogrel bisulfate 75mg once a day.

**Figure 2:**
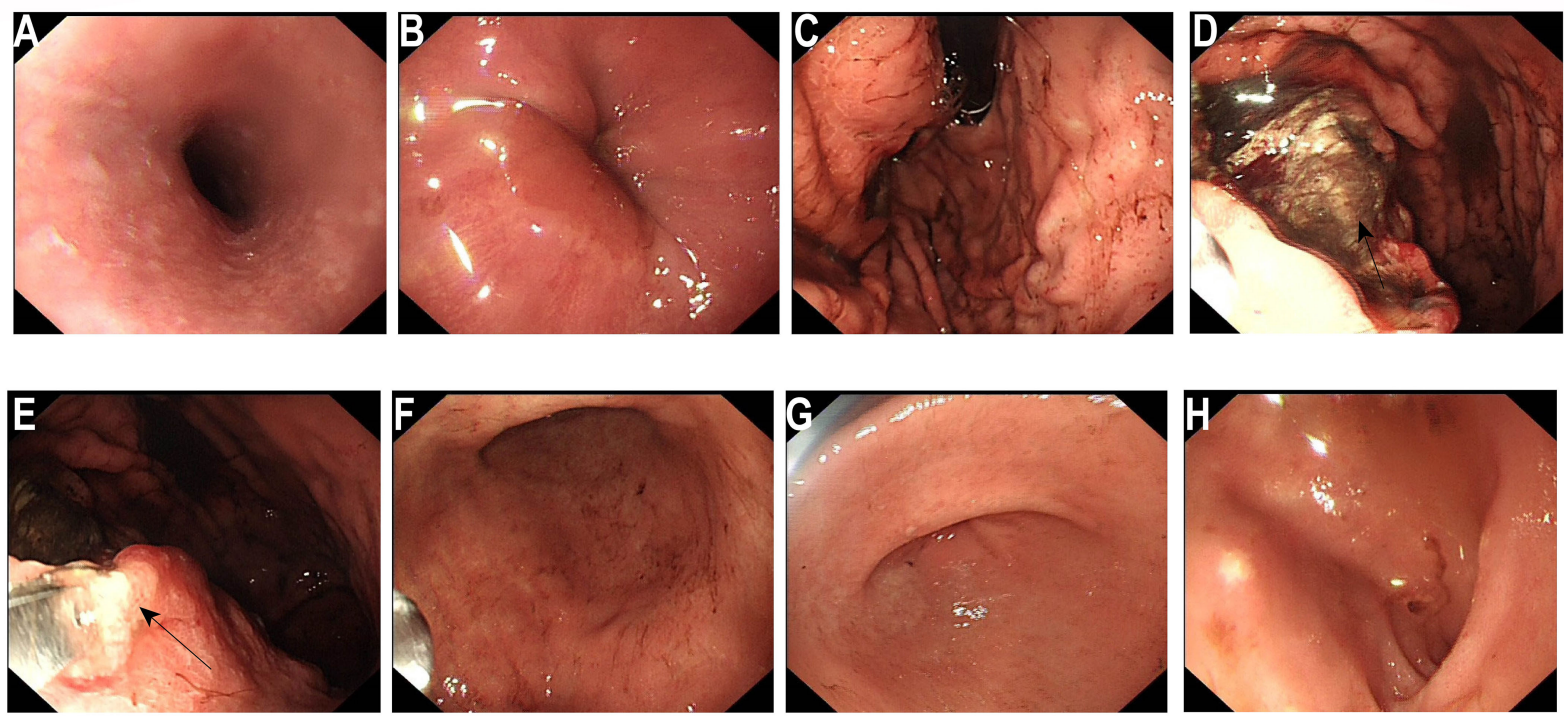
Initial electronic gastroscopy. **(A)** Esophagus: The mucosa is smooth and soft, with clear vascular patterns. **(B)** Cardiac valve: dentate line is clear. **(C)** Gastric fundus: The gastric fundus contains coffee-ground material. **(D)** A huge irregular ulcerative change can be seen on the posterior wall (←), covered with black blood and a small amount of fresh bloodstains. The surrounding mucosa is edematous and rough. Biopsy specimens were taken from two areas at the edge of the ulcer (←). The mucosa of the lesser curvature and the anterior and posterior walls of the stomach is pale, extending to the outside of the cardia. **(E)** Gastric angle: Arc present, the mucosa appears pale (←). **(F)** Gastric antrum: Mucosal red and white alternating, pale mucosal range increased. **(G)** Pylorus: Visible, round in shape, opening and closing freely. **(H)** Duodenum: No abnormalities were found in the duodenal bulb and descending part.

During the course of inpatient treatment, in order to further clarify the nature of the space-occupying lesion in the gastric body, the patient underwent the first gastroscopy on June 25, 2025 (**Figure 2**). The endoscopic observations were as follows: 1. A massive ulcerative lesion accompanied by bleeding in the gastric body; 2. Atrophic gastritis (considered based on endoscopic findings). Due to active bleeding, endoscopists only obtained biopsies from two superficial apparent lesions and did not attempt more extensive sampling.

**Figure 3:**
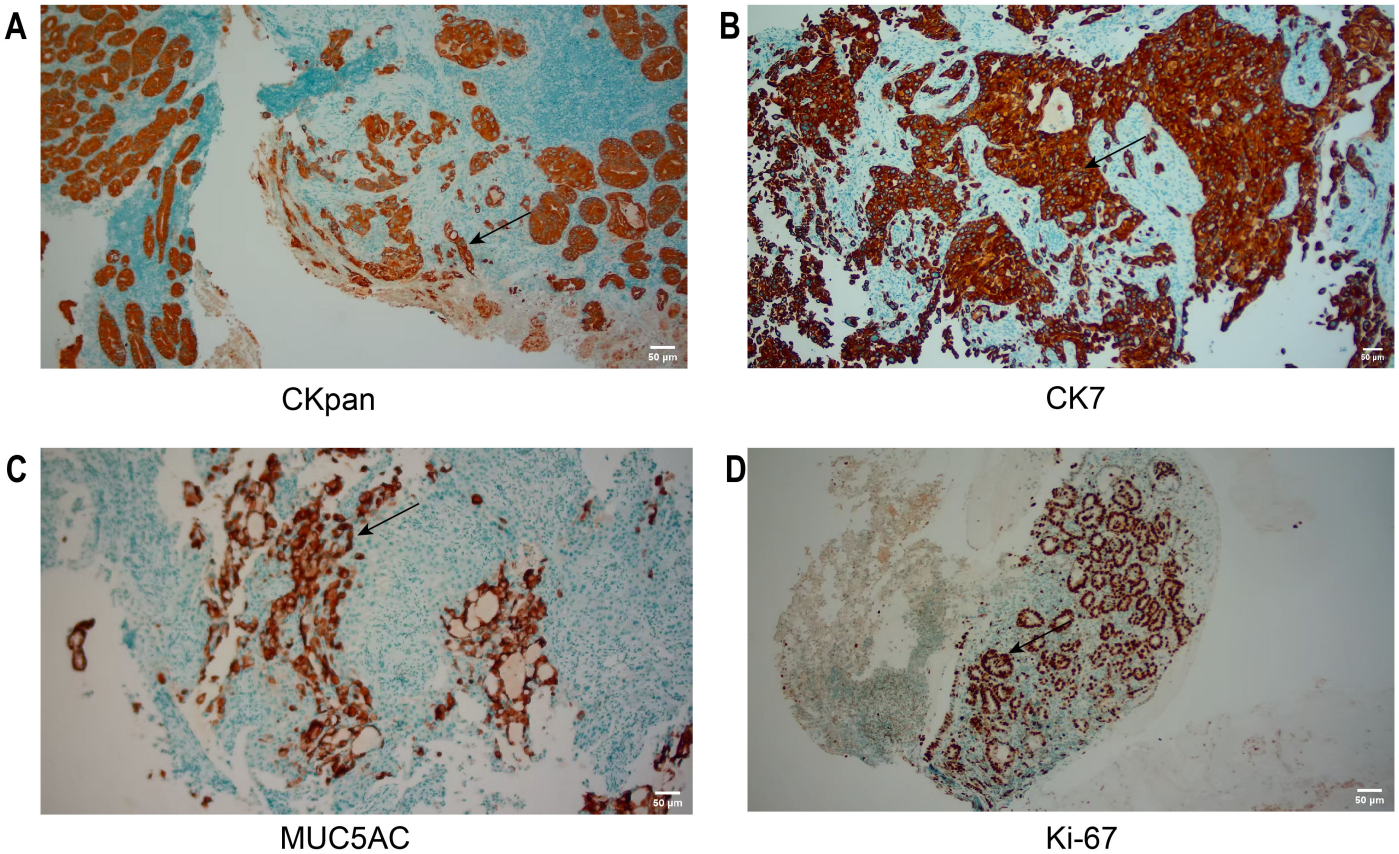
IHC of the initial biopsy. **(A, B)** CKpan, CK7: Tumor cells were diffusely positive. **(C, D)** MUC5AC, Ki-67: Tumor cells were focally positive. The area indicated by the arrow is the region of positive expression. Scale bar: 50 μm (applicable to all panels).

On June 27, 2025, rapid paraffin section examination and diagnosis were conducted: (Posterior wall of the gastric body) Chronic ulcer, with an activity level of ++. A small number of glandular structures were found to be disorganized at the edge of one of the mucosal samples, and the cellular atypia was relatively prominent. Therefore, adenocarcinoma could not be ruled out. Immunohistochemical results (**Figure 3**) were as follows: CKpan (+), CK7(+), MUC5AC (focally +), Ki-67 (partially +). There was a tendency towards gastric adenocarcinoma invasion.

Following a consultation with the Department of Gastrointestinal Surgery, taking into account the patient’s and her children’s opinions, as well as factors such as the scope of tumor invasion, which included the gastric body, splenic artery, and pancreas, and the obvious arterial invasion, the following recommendation was put forward: A comprehensive assessment determined that surgery was not feasible. Regarding the patient’s concurrent splenic infarction, the decision of whether to perform emergency or elective surgery would be based on the presence or absence of symptoms, and no immediate intervention was required for the time being.

**Figure 4:**
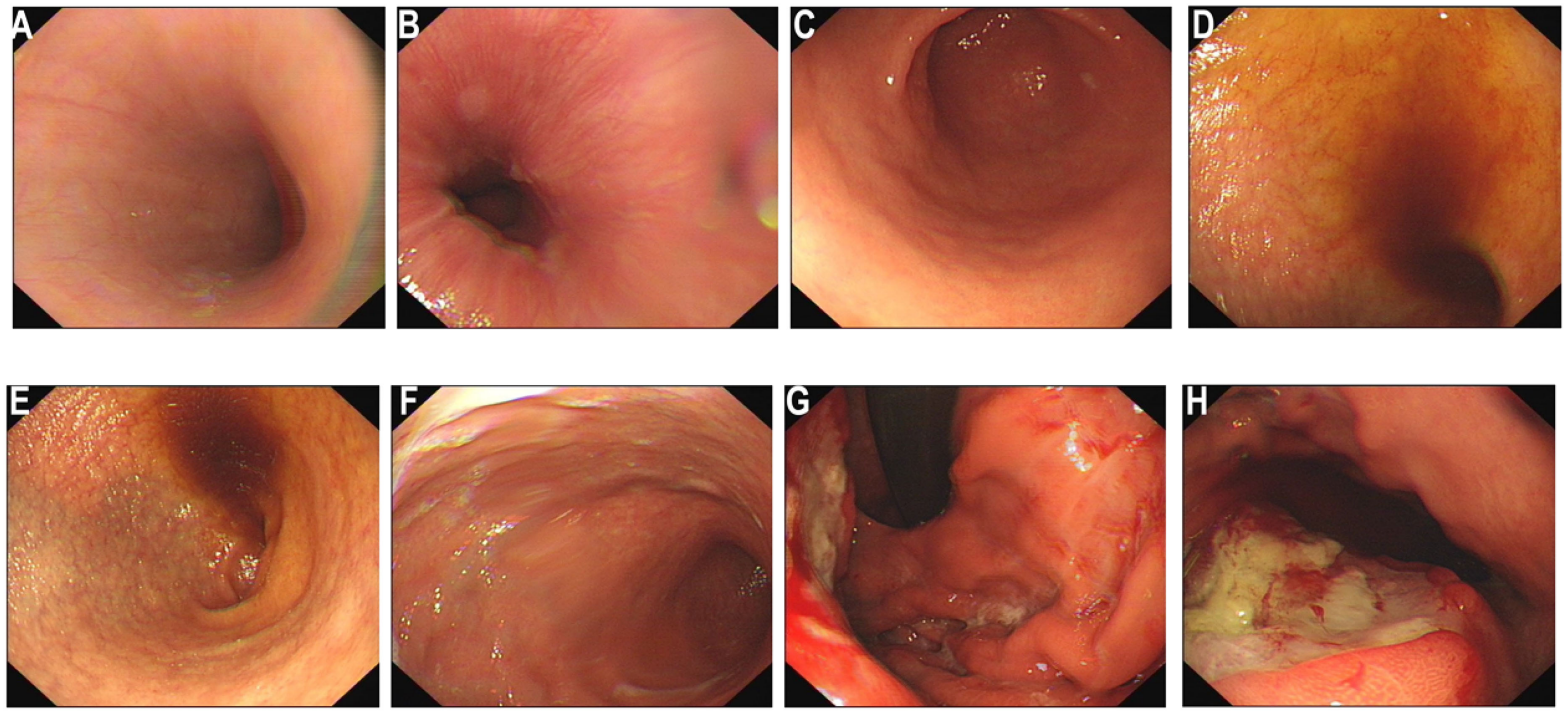
Electronic gastroscopy again. **(A–F)** Endoscopic findings of the esophagus, cardia, body and antrum of the stomach: The esophageal mucosa is smooth with normal vascular patterns; the cardia mucosa shows mild congestion; no obvious abnormal lesions are observed in the antrum. **(G, H)** A large ulcerative neoplasm on the posterior wall of the gastric body (black ←) was re-biopsied for pathology ×6 pieces. Diagnosis: Gastric space-occupying lesion (nature to be determined by pathology).

**Figure 5:**
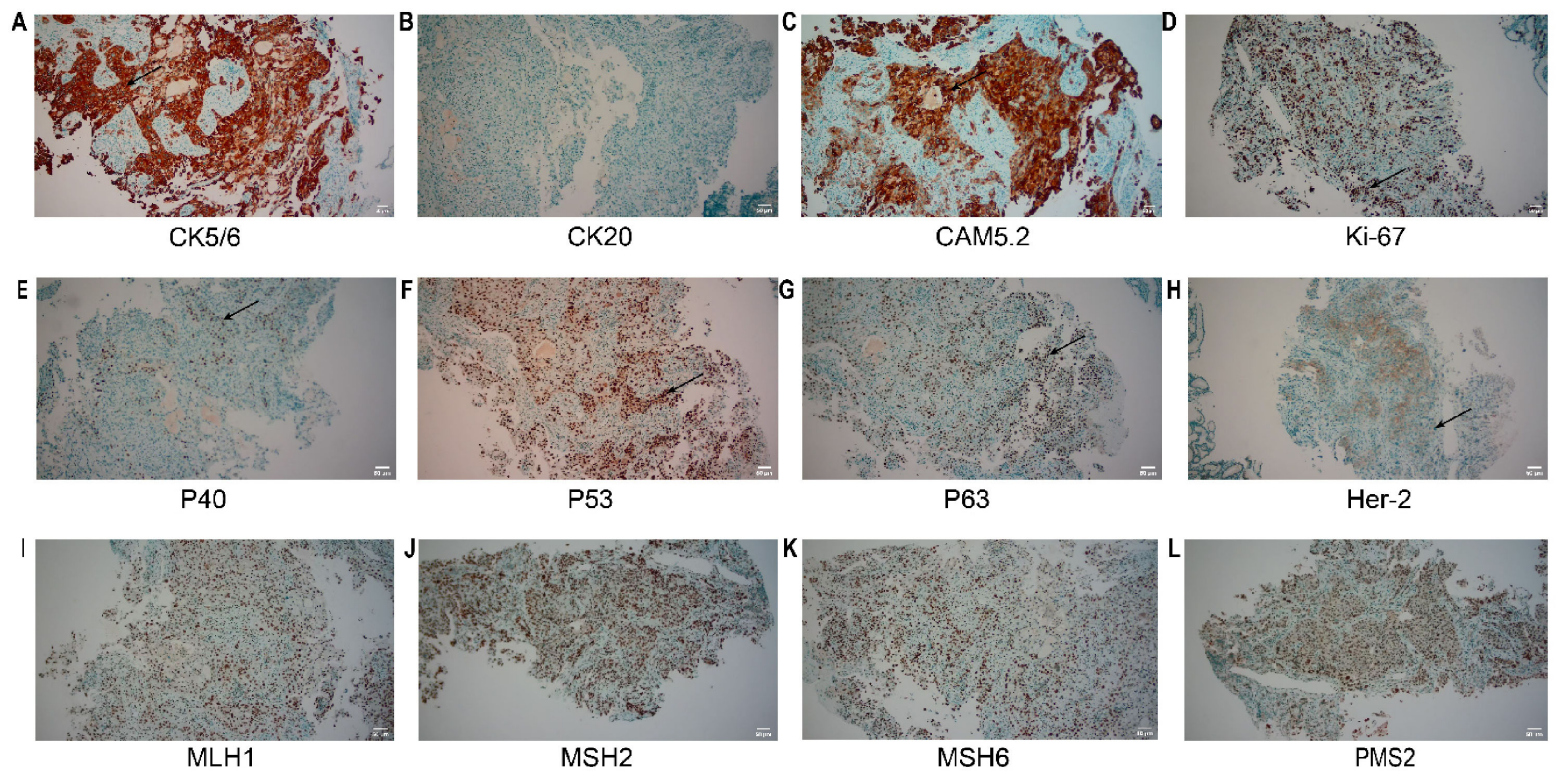
IHC of the re-biopsy. **(A)** CK5/6: Tumor cells show diffuse cytoplasmic/nuclear positivity; **(B)** CK20: Tumor cells are negative; **(C)** CAM5.2: Tumor cells show diffuse strong positivity; **(D)** Ki-67: The tumor cell proliferation index is approximately 60%, with nuclear positivity; **(E)** P40: Tumor cells show nuclear positivity; **(F)** P53: Tumor cells show nuclear positivity, consistent with mutant expression; **(G)** P63: Tumor cells show nuclear positivity; **(H)** Her-2: Tumor cells show 2+ expression; **(I-L)** MLH1, MSH2, MSH6, PMS2: Tumor cells all show nuclear positivity, suggesting intact protein expression. The area indicated by the arrow is the region of positive expression. Scale bar: 50 μm (applicable to all panels).

For further treatment, the patient was transferred to the oncology department. The patient’s family requested prompt initiation of treatment based on the pathological findings suggestive of gastric adenocarcinoma. Considering that the biopsy material obtained during the gastroscopy was limited and only highly suggested a diagnosis of gastric adenocarcinoma without a definitive confirmation, we repeatedly emphasized the necessity of a second gastroscopy to the patient’s family. On July 3, 2025, the second gastroscopy was performed (**Figure 4**). The rapid paraffin section examination and diagnosis of the gastroscopy indicated: (posterior wall of the upper part of the gastric body, biopsy) poorly differentiated malignant tumor; combined with immunohistochemical staining, it was consistent with squamous cell carcinoma. Immunohistochemistry (**Figure 5**): CK5/6 (+), P40 (+), CK20 (-), MUC5AC (local +), Cam5.2 (+), CK7 (+), P63 (+), P53 (+, mutant type expression), HER-2 (2+), MLH1 (normal expression), PSM2 (normal expression), MSH2 (normal expression), MSH6 (normal expression), HP (-), Ki-67 (+, 60%). Finally, based on CT and pathology, the clinical diagnosis was gastric squamous cell carcinoma cT4bN2M0 stage IVA.

Considering the unclear expression status of genes such as PD-L1, Her-2, and Claudin 18.2 in the patient, we suggested conducting relevant gene testing. However, the patient’s family declined further genetic testing. After a comprehensive assessment of the patient’s overall condition and a multidisciplinary team (MDT) consultation, treatment with Raltitrexed (4mg) and Sintilimab (200mg) was initiated for two cycles. The patient tolerated the treatment well, and is currently under follow-up.

## 3 Discussion

Primary gastric squamous cell carcinoma (PGSCC) is a highly challenging malignant tumor, with diagnosis and treatment fraught with pitfalls and uncertainties. This case report describes the diagnostic and therapeutic course of a 77-year-old female with PGSCC in the gastric body (not at the esophagogastric junction), profoundly highlighting these challenges. Gastric body primary squamous cell carcinoma rules out the possibility of esophageal origin invading the esophagogastric junction, and its epidemiological significance is even rarer. Here, we will conduct an in-depth discussion from four dimensions: diagnosis, pathogenesis, treatment strategies, and future prospects, in combination with the latest literature.

### 3.1 Review and in-depth analysis of diagnostic traps

The diagnostic trajectory in this case is illustrative. The first biopsy, due to the limited tissue sample, could only provide a vague diagnosis of “possible adenocarcinoma” based on CKpan (+), CK7(+), MUC5AC (focal+), and Ki-67(partly+), which highlights a common but underappreciated issue in current clinical practice: overreliance on the diagnostic value of limited specimens.

Recent studies have found that the immunohistochemical manifestations of poorly differentiated gastric cancer show significant lineage ambiguity. In 2023, Takada et al. published a single-cell sequencing study in Nature Communications, revealing that the differentiation mechanisms of gastric epithelial cells (such as pit cells, neck cells, and parietal cells) may involve multiple signaling pathways including EGFR, BMP, SHH, IGF, and specific transcription factors such as PPARγ, Pbx1, and Ybx2 (9). Additionally, in 2023, Chinese scholars Tang et al. discovered that genes maintaining normal gastric epithelium and function, such as MUC6, and genes related to gastric cancer development, such as MUC13, CLDN3/4, show significant differences between pathological types, whether intestinal or mixed gastric cancer (10). This molecular-level finding accounts for the focal positivity of adenocarcinoma markers such as MUC5AC and CK7 in the setting of insufficient tissue sampling where tumor heterogeneity is not captured, potentially leading to misdiagnosis. Therefore, making a tendency diagnosis based on limited immunohistochemical markers from a small tissue sample carries a very high risk (11, 12). In the present case, focal positivity of CK7 and MUC5AC was initially detected by immunohistochemistry, which led to the misdiagnosis of gastric squamous cell carcinoma. This phenomenon is not an individual case, but the result of the joint action of multiple factors, including tumor heterogeneity, abnormal differentiation, and sampling limitations (13).

Tumor heterogeneity is an important feature of tumors, and tumor cells in different regions may exhibit different molecular phenotypes and degrees of differentiation (14). In esophageal squamous cell carcinoma, studies have found the expression of the adenocarcinoma marker AGR2, which reflects the abnormal protein expression caused by tumor heterogeneity (15). Similarly, in gastric squamous cell carcinoma, focal positivity of adenocarcinoma related markers may also occur because of tumor heterogeneity. This heterogeneity means that a single biopsy may not fully capture the true characteristics of the tumor, leading to diagnostic bias.

Abnormal differentiation is also an important factor leading to the expression of atypical markers (16). MUC5AC is a secreted mucin, which is mainly expressed in normal gastric mucosa, barrett’s esophagus and adenocarcinoma. However, studies have shown that focal positivity of MUC5AC may also occur in a few squamous cell carcinomas (17). This indicates that tumor cells may have undergone abnormal differentiation and expressed some proteins that usually do not belong to their lineage. This abnormal differentiation may also occur in gastric squamous cell carcinoma, leading to focal positivity of adenocarcinoma related markers such as MUC5AC.

In addition, sampling limitations are also an important reason for diagnostic bias. In this case, the first endoscopic biopsy only took superficial tissue, and the sample size was insufficient due to active bleeding, which may lead to tissue fragmentation or only obtain necrotic/inflammatory areas. This small sample has high uncertainty in diagnosis, especially in elderly patients, which may be more likely to cause the deviation in the interpretation of immunohistochemical results (18). More complete tissue samples were obtained from subsequent deep biopsies to confirm the expression of squamous cell carcinoma markers.

Our case was successfully diagnosed through a second deep biopsy, which not only validates the principle of “adequate sampling” but also refines it. The 2024 CSCO guidelines and expert consensus on gastric cancer clearly state that for difficult cases, biopsies should aim to capture the heterogeneity within the tumor rather than merely identifying malignant cells. It is recommended to use forceps or large biopsy forceps to take samples from multiple sites and depths around the ulcer margin, base, and surrounding mucosa to obtain sufficient tissue for comprehensive lineage analysis including MMR, PD-L1, and even NGS (19–22).

### 3.2 Reconsideration of the origin and microenvironment of gastric squamous cell carcinoma

The discovery of atrophic gastritis under gastroscopy in this case provides a unique perspective for exploring the mechanism of tumor development. Atrophic gastritis is a classic precancerous lesion of gastric adenocarcinoma (23, 24), but its association with PGSCC has always been controversial. Recent studies tend to suggest that the immunosuppressive and pro-inflammatory microenvironment created by chronic atrophic gastritis, characterized by the continuous high expression of cytokines such as IL-6 and TNF-α, may be a more upstream driving factor. This microenvironment can induce epithelial-mesenchymal transition (EMT) in mucosal epithelial cells and reduce genomic stability, thereby providing a “soil” for various types of carcinogenesis, including both adenocarcinoma and squamous cell carcinoma (25–28).

The TP53 mutant expression and high Ki-67 index found in this case are key molecular events worthy of in-depth analysis. TP53 mutations are genetic alterations present in the development of various tumors, leading to cell cycle dysregulation and genomic instability, and act as accelerators of tumorigenesis (29, 30). Recent studies have shown that in the context of chronic inflammation, TP53 mutations may cooperate with other driver events (such as the loss of EZH2 and Pten genes) to force damaged gastric stem cells to escape towards a squamous differentiation pathway, ultimately leading to the formation of PGSCC (16). Therefore, the atrophic gastritis in this case should be regarded more as a background disease that creates a carcinogenic “soil”, while events such as TP53 mutations are the “seeds” sown within it.

### 3.3 The evolution of treatment strategies

Although PGSCC is rare, numerous large-scale clinical studies on gastric cancer using chemotherapy combined with immune checkpoint inhibitors have confirmed their effectiveness (such as CheckMate 649, ATTRACTION-4, KEYNOTE-859, etc.) (16, 31, 32), and the molecular characteristics of PGSCC suggest that it may be sensitive to immunotherapy. In this case, due to the patient’s advanced age, poor general condition, and refusal of surgery, we referred to the ORIENT-16 trial protocol (33). Raltitrexed, a specific thymidylate synthase inhibitor, was selected primarily due to its more favorable safety profile, particularly its lower incidence of gastrointestinal and hematologic toxicity compared to fluoropyrimidines, making it a suitable option for this elderly patient with potential organ function compromise (34, 35). Finally, Raltitrexed was combined with the PD-1 inhibitor Sintilimab, in a regimen designed to balance efficacy and tolerability. This decision was endorsed by the multidisciplinary team, which considered the patient’s advanced age, poor performance status, and refusal of surgery.

Furthermore, this case underscores the pivotal role of a multidisciplinary team (MDT) in managing such complex patients. Beyond formulating anti-tumor strategies, the MDT should comprehensively address patients’ supportive care needs. Particularly for elderly patients with advanced gastric cancer presenting with symptoms such as abdominal pain and bleeding, there is a prevalent risk of malnutrition and cachexia (36). Therefore, integrating a clinical nutritionist into the MDT to provide professional nutritional assessment and intervention is crucial for improving treatment tolerance, maintaining quality of life, and optimizing overall clinical outcomes (36).The patient only experienced grade I gastrointestinal reactions and tolerated the treatment well (33).

For the operable patients, in 2024, Cheng et al. retrospectively analyzed the data of 334 cases of GSCC from the SEER database and found that the 5-year overall survival rate (32.07%) and tumor-specific survival rate (41.93%) of the surgical group were significantly better than those of the non-surgical group (11.08% and 18.45%). Marital status, pathological differentiation, etc. were independent prognostic factors for GSCC. Married patients, those with well-differentiated tumors, and those who received radiotherapy had the best surgical benefits (37).

This case highlights the diagnostic pitfall of inadequate initial biopsy for the diagnosis of primary gastric squamous cell carcinoma. Focal positivity for adenocarcinoma markers such as CK7, MUC5AC in small specimens can be misleading due to the presence of tumor heterogeneity. Therefore, the diagnosis must be confirmed by deep, multi-site biopsy combined with complete immunohistochemical test including squamous markers such as P40 and CK5/6, following the principle of “binary exclusion method”. The atrophic gastritis found under endoscopy in this case provides clues to its pathogenesis, and the chronic inflammatory microenvironment caused by it may serve as the “soil” (38), which may promote the transformation of gastric mucosa into squamous differentiation pathway under the cooperation of key driver gene mutations such as TP53 (39). In terms of treatment, given the patient’s advanced age, poor performance status, and refusal to undergo surgery, we chose a palliative regimen of “Sintilimab plus Raltitrexed”. PD-1 inhibitor Sintilimab was selected based on the possible sensitivity of squamous cell carcinoma to immunotherapy and the evidence of ORIENT-16 et al. in gastric cancer. The choice of Raltitrexed is mainly due to its better tolerance than fluorouracil in elderly patients. Although HER2 testing was 2+ in this case, its clinical significance was unclear in the absence of FISH confirmation, which suggests the potential value of routine comprehensive molecular profiling of PGSCC but is not central to this individualized treatment decision.

### 3.4 Limitations and future directions

This study, as a detailed case report, has limitations due to its single-center experience and relatively short follow-up period. However, it accurately reflects the core pain points in the current diagnosis and treatment of PGSCC: insufficient awareness, lack of diagnostic criteria, and scarcity of treatment evidence. The future directions of efforts should be:

1. Establishing an international registry system: By collecting clinical, pathological, and molecular data of PGSCC cases on a large scale through international multi-center collaboration, a foundation for truly understanding this disease can be laid.
2. Defining molecular subtypes: Utilizing multi-omics technologies, attempts should be made to classify PGSCC into different molecular subtypes (such as immune prototype, stem cell type, classic squamous cell carcinoma type, etc.), thereby tailoring treatment strategies for different subtypes.
3. Exploring cutting-edge therapies: Given its rarity, priority should be given to exploring basket trial designs, including PGSCC in new drug clinical trials based on biomarkers (such as high TMB, specific gene mutations) along with squamous cell carcinomas in other sites such as the head and neck and esophagus.

## 4 Conclusions

This case report provides a detailed account of the entire diagnosis and treatment process of a squamous cell carcinoma (SCC) in the gastric body in an elderly female. This case highlights the core challenges in the diagnosis of primary gastric squamous cell carcinoma (PGSCC): the initial biopsy is prone to misdiagnosis due to insufficient tissue volume and tumor heterogeneity. The diagnosis must follow the principle of the “binary exclusion method” of immunohistochemistry and obtain definite pathological evidence through adequate deep sampling.

At the treatment level, immunotherapy combined with chemotherapy based on molecular features (such as PD-L1 and TMB) offers a promising treatment option for elderly patients who are not suitable for surgery. The results of this study support the routine molecular testing of PGSCC and emphasize the establishment of an international case registration system to collect more clinical and molecular data as a key path to improving the prognosis of this rare disease.

## Data Availability

The original contributions presented in the study are included in the article/Supplementary Material. Further inquiries can be directed to the corresponding author.
